# Surface modification of carbon-based adsorbents derived from coal gasification slag for the efficient adsorption of trimethoprim

**DOI:** 10.1371/journal.pone.0351221

**Published:** 2026-06-10

**Authors:** Zhiqiang Yang, Jia Liu, Qingchao Li

**Affiliations:** 1 Xinjiang Tianye Huihe New Material Co., Ltd., Shihezi, Xinjiang, China; 2 College of Chemistry and Chemical Engineering, Xi’an University of Science and Technology, Xian, Shanxi, China; 3 Ningbo Antai Environmental Chemical Engineering Design Co., Ltd., Ningbo, Zhejiang, China; University of Tehran, IRAN, ISLAMIC REPUBLIC OF

## Abstract

Given that pristine water bodies are irreplaceable for maintaining ecosystem homeostasis, the remediation of antibiotic-contaminated aquatic systems has emerged as a pivotal challenge. This research used flotation residual carbon (FRC) obtained from coal gasification coarse slag (CGCS) as a precursor and adopted the H_2_O_2_ liquid-phase oxidation method to prepare a low-cost and efficient adsorbent by optimizing process parameters such as modification temperature, time, and oxidant concentration. The H_2_O_2_ treated flotation residual carbon was denoted as HFRC. Batch adsorption assays revealed that, under their respective optimal conditions, equilibrium uptakes of Trimethoprim (TMP) by FRC and HFRC reached 54.02 mg/g and 80.01 mg/g, with equilibrium times of 270 min and 360 min. Comprehensive characterization, molecular simulations, and model fitting collectively elucidated the adsorption mechanism and the kinetic intensification of TMP. H_2_O_2_ modification markedly increased the abundance of oxygen-containing functionalities. On this basis, the van der Waals and electrostatic interactions between HFRC and TMP were significantly enhanced, accompanied by a pronounced increase in hydrogen-bond density, which collectively accelerated the TMP adsorption kinetics. HFRC also exhibited exceptional dynamic adsorption performance in the fixed-bed column. Overall, HFRC integrates rapid removal performance with resource-cycling benefits and superior environmental adaptability, offering an economically and ecologically synergistic paradigm for the valorization of CGCS and the remediation of antibiotic-impaired waters.

## 1 Introduction

Population growth, increased life expectancy, and improved healthcare have led to an exponential rise in the clinical and agricultural usage of antibiotic emerging contaminants of concern (CECs) such as trimethoprim (TMP) [1]. Following excretion, these compounds are continuously discharged into water bodies via municipal wastewater and agricultural runoff. Owing to their structural stability and high biological activity, they accumulate at the sediment-water interface, with concentrations potentially exceeding the μg/L threshold [2,3]. Trophic magnification disrupts the gut microbiome, facilitates horizontal gene transfer of antibiotic-resistance determinants, and imposes oxidative stress on plant roots, ultimately impairing carbon fixation by primary producers [4,5]. Consequently, proactive “source-to-receptor” control strategies that achieve efficient and sustainable elimination of pharmaceutical residues before they reach aquatic environments are urgently required to safeguard ecosystem health [6].

Among available technologies, adsorption is widely recognized as a core strategy for remediating contaminated waters because of its operational simplicity, low energy demand, and high removal efficiency [7,8]. Consequently, a range of advanced sorbents-activated carbons, magnetic chitosan-Fe_3_O_4_ nanocomposites, functionalized silica chelators, graphene oxides, natural porous minerals, and biochar-have been developed. However, it has been observed that each material exhibits intrinsic structure-performance bottlenecks under field conditions [9]. Magnetic chitosan-Fe_3_O_4_ is highly pH-sensitive; acidic or alkaline media markedly reduce its capacity, and the chitosan backbone is susceptible to microbial degradation, necessitating costly preservatives for long-term storage [10]. Functionalized silicas offer excellent selectivity, but multi-step syntheses escalate costs and their surface protonation diminishes at pH > 8, demanding tight pH control around 8 to maintain performance [11]. Activated carbons suffer from rate-limiting micropore blockage by natural organic matter; thermal or chemical regeneration is energy-intensive and carbon-depletive, undermining economic viability [12]. Graphene oxides readily aggregate in real waters and require magnetic dopants or polymeric stabilizers; their fabrication involves strong oxidants that pose secondary environmental risks [13]. Natural porous adsorbents are constrained by high acid, energy, and water consumption together with operational safety issues, limiting their scalability [14]. Collectively, current adsorbents fail to reconcile capacity, selectivity, stability, cost-effectiveness, and ecological safety. Biochar is typically produced through pyrolysis, involving thermal treatment at elevated temperatures (commonly ranging from 350 °C to 700 °C) under oxygen-limited or anoxic conditions [15]. There is therefore an urgent need for next-generation materials that efficiently remove TMP while meeting the dual imperatives of environmental protection and human-health security [3,16]. Moreover, current simulations of antibiotic adsorption have predominantly focused on the natural population analysis charges and surface electrostatic potential distributions of adsorbents and antibiotics [17], the total adsorption energy variations associated with different functional groups on the adsorbent [18], the preferential occupation of adsorption sites by antibiotic molecules [19], the adsorption trajectories within idealized pore models of hierarchical porous carbons [20], and the adsorption behaviors on adsorbents with varied interlayer spacings [21]. Notably, dynamic simulations that capture the time-resolved adsorption pathway of antibiotic molecules onto chemically modified adsorbent surfaces remain scarce; consequently, the temporal evolution of adsorption routes, conformational transitions, and intermolecular interactions is poorly understood. This absence of atomistic, time-dependent insight constrains the formulation of realistic theoretical frameworks necessary for elucidating the underlying adsorption mechanisms.

The annual production of coal gasification slag (CGS) is substantial, exceeding 50 million tons [22,23]. After classification and separation, the residual carbon (RC) obtained is a by-product characterized by both high carbon content and a well-developed porous structure. This renders it highly advantageous for resource utilization in adsorption and separation applications [24,25]. RC inherits a rich meso- and microporous system formed during the gasification process, which facilitates the diffusion of large organic molecules while ensuring high-capacity adsorption of trace heavy metal ions via micropores [26,27]. The surface of RC contains abundant functional groups such as carboxyl, phenolic hydroxyl, and aromatic rings, enabling stable complexation with organic pollutants through multiple mechanisms including electrostatic attraction, complexation, and hydrogen bonding [28,29]. Due to the partial graphitization that occurs during the high-temperature (>1000 °C) gasification and quenching process, RC exhibits superior thermal shock resistance and compressive strength compared to traditional biomass-derived adsorbents [25,30]. Previous studies have demonstrated that CGS-based adsorbents show promising performance in CO_2_ capture [31], organic pollutant removal [32], and metal ion recovery [33]. However, systematic investigations into the adsorption behavior of TMP remain scarce. Conventional modification strategies such as acid leaching, calcination, and alkaline treatment are often constrained by harsh reaction conditions, high energy consumption, and severe equipment corrosion, making them incompatible with the principles of green chemistry [29]. Therefore, there is an urgent need to develop efficient, low-energy, and environmentally friendly surface functionalization techniques to enhance the TMP adsorption performance of CGS. H_2_O_2_ modification offers a promising alternative, enabling the preservation of adsorbent structural integrity under mild conditions while achieving high-density introduction of surface oxygen-containing functional groups, controllable optimization of pore structures, and precise regulation of surface charge [34,35]. Legocka et al. [34] employed H_2_O_2_-oxidized carbon black for the elimination of 2,4-dichlorophenoxyacetic acid and 2-methyl-4-chlorophenoxyacetic acid. Peña et al. [35] utilized H_2_O_2_ to tailor the surface chemistry and pore architecture of glycerol-derived porous carbons, thereby enhancing CO_2_ uptake capacity. Zhang et al. [36] synthesized H_2_O_2_-modified peanut shell biochar/sodium alginate microspheres for the high-efficiency sequestration of Cu(II). Thue et al. [37] functionalized natural montmorillonite clay with H_2_O_2_ to enrich surface moieties and achieve superior removal of Acid Red 114. Badawy et al. [38] incorporated H_2_O_2_ during the fabrication of black clay/rice flour/Fe_3_O_4_ (BC/RF/MNPs) adsorbents, resulting in advanced performance in ibuprofen abatement. However, research on the surface modification of RC from CGS using hydrogen peroxide remains limited. Its potential for the removal of antibiotic pollutants such as TMP warrants further investigation, and molecular simulation methods for the adsorption process have been underutilized.

CGS can be taxonomically differentiated into two distinct fractions: coal gasification coarse slag (CGCS) and coal gasification fine slag (CGFS) [22]. In this study, flotation-enriched residual carbon (FRC) derived from CGCS was employed as a substrate and subjected to mild oxidative modification using H_2_O_2_. The effects of modification temperature, reaction time, and H_2_O_2_ concentration on the adsorption performance of FRC were systematically investigated. Comprehensive characterization, molecular simulations, and model fitting were employed to elucidate the differences in TMP adsorption mechanisms before and after modification. This approach enabled the low-cost, low-energy, and clean preparation of an efficient TMP adsorbent, offering dual benefits in environmental protection and public health.

## 2 Materials and methods

### 2.1 Materials

**Fig 1:**
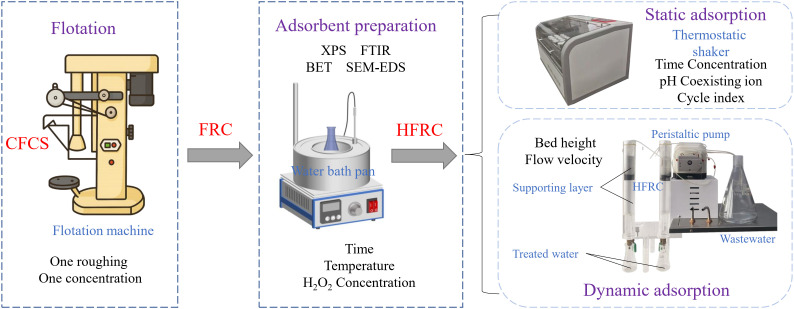
Schematic diagram of adsorbent preparation and testing process.

In this study, the CGCS was sourced from Xinjiang Province, China. The schematic illustration of both the preparation protocol and the experimental adsorption procedure is presented in Fig 1. The fraction below 0.5 mm (ash content 60.12%) was employed as feed for foam flotation recovery of RC (Tables 1 and 2). A 100 g aliquot of the prepared CGCS was slurried with 1 L tap water in a flotation cell; the impeller was rotated at 1800 rpm for 2 min to condition the pulp. Subsequently, 14 kg/t kerosene was introduced as collector and agitated for 2 min, followed by 10 kg kg/t sec-octyl alcohol as frother with an additional 2 min of conditioning. The air valve was then opened to initiate flotation, which was performed twice [39]. During the concentrating stage, the dosages of collector and frother were reduced to 2.8 and 2 kg/t, respectively. The concentrate (i.e., flotation residual carbon, designated as FRC) had an ash content of 11.24% (Table 2) and was subsequently subjected to surface modification using H_2_O_2_. TMP (≥98.0%) used to prepare the synthetic pharmaceutical pollutant solution was purchased from Shanghai Aladdin Biochemical Technology Co., Ltd.; H_2_O_2_ (analytical grade) was supplied by Sinopharm Chemical Reagent Co., Ltd.

**Table 1:** Flotation results of CGCS.

Products	Yield	Ash content	Cumulative yield	Cumulative ash content
Concentrate	19.13	11.26	19.13	11.26
Middlings	6.33	30.87	25.46	16.14
Tailings	74.54	75.09	100.00	60.08
Total	100.00	60.08		

**Table 2:** Proximate and ultimate analysis of Sample.

Sample	M_ad_	A_ad_	V_ad_	FC_ad_	C_ad_	H_ad_	N_ad_	O_ad_	S_t,d_
CGCS	0.97	60.12	3.10	35.81	36.19	0.41	0.29	1.67	0.37
FRC	0.25	11.24	3.58	84.93	84.91	0.50	0.73	0.76	0.48

The following definitions are provided on an air-dried basis: M_ad_ denotes the moisture content, i.e., the mass fraction of inherent water present in the sample under air-dried conditions; A_ad_ is the ash content, representing the mass fraction of inorganic residue remaining after complete combustion of the sample; V_ad_ refers to the volatile matter, i.e., the mass fraction of gaseous substances released when the sample is heated in an oxygen-free atmosphere; FC_ad_ is the fixed carbon, corresponding to the combustible solid carbon in the sample; and C_ad_, H_ad_, N_ad_, and O_ad_ represent the mass fractions of carbon, hydrogen, nitrogen, and oxygen elements, respectively. Finally, S_t,d_ is the total sulfur on a dry basis (free from moisture), defined as the total mass fraction of all sulfur species (organic sulfur, pyritic sulfur, and sulfate sulfur) in the sample.

### 2.2 Adsorbent preparation

A precise quantity of 10g of FRC was transferred to a 100-mL Erlenmeyer flask. The flask was then treated with 60 mL of H_2_O_2_ solution, the mass fraction of which had been previously determined. The contents of the flask were agitated at 150 rpm for a fixed duration at a set temperature. The solid was recovered by vacuum filtration, washed with distilled water until neutrality, dried at 105 °C, and stored in a sealed vial to yield the modified FRC. Triplicate runs were performed; the adsorption capacities toward TMP were averaged.

### 2.3 Test characterization

Phase identification was performed on a SmartLab X-ray diffractometer (XRD) (Rigaku, Japan). Surface chemical bonds and functional groups were analysed with a Nicolet Summit X FTIR spectrometer (Thermo Fisher Scientific, USA). Elemental compositions and surface stoichiometries were determined by X-ray photoelectron spectroscopy (XPS) (ESCALAB 250Xi, Thermo Fisher Scientific) calibrated to the C 1s peak at 284.8 eV. Morphological features were visualised with a Verios XHR scanning electron microscope (SEM) (Thermo Fisher Scientific). Pore-size distributions and specific surface areas were measured by N_2_ physisorption at −196 °C using an ASAP 2460 automated analyser (Micromeritics, USA); samples were out-gassed at 200 °C and 100 mmHg for 600 min with a heating ramp of 10 °C/min.

### 2.4 Molecular simulation

Quantum chemical calculations were performed to investigate the fundamental properties of TMP, oxygen-containing functional groups, and benzene rings. Molecular models were constructed using the Visualizer module of Materials Studio 6.0, and subsequent calculations were conducted with the Dmol3 module. The computational parameters were as follows [40]: the exchange-correlation energy was described by the generalized gradient approximation (GGA-PW91), and the double numerical basis set including polarization functions (DNP) was employed. The self-consistent field (SCF) convergence criterion was set to 10^−5^, while the convergence thresholds for energy, maximum force, and maximum displacement were 2 × 10^−5^ Ha, 4 × 10^−3^ Ha/Å, and 5 × 10−^3^ Å, respectively.

To further elucidate the adsorption behavior of TMP on various surface functional groups of the adsorbent from a microscopic perspective, molecular simulations were conducted using the Forcite module of Materials Studio 6.0 [41]. Oxygen-containing functional groups (C-O-C, C = O, -COOH, and -OH) were grafted onto the graphite model surface, and the initial configuration of the adsorbent, TMP, and vacuum layer was constructed with dimensions of 50 Å × 50 Å × 87 Å. The COMPASS force field was adopted for the simulations. Structural optimization of the models was performed using the Smart method in the Geometry Optimization module with 20,000 iterations. Molecular dynamics simulations were then carried out under an NVT ensemble at 298.0 K, with temperature control achieved via the Nose thermostat and a time step of 1.0 fs. Electrostatic interactions were calculated using the Ewald method with an accuracy of 0.0001 kcal/mol, while van der Waals interactions were treated using the atom-based method with a cutoff radius of 15.5 Å. All systems reached equilibrium after 500 ps, and the results were subsequently analyzed [42].

### 2.5 Static adsorption

In the context of the adsorption assays, a quantity of 0.03g of adsorbent was introduced into 50 mL of TMP solution. The pH was adjusted to the desired value with dilute NaOH or HCl. The suspension was transferred to a thermostatic shaker and equilibrated at 25 °C. After predetermined intervals, 10 mL aliquots were withdrawn with a syringe and immediately filtered through a 0.22 μm membrane. The residual TMP concentration was quantified by high-performance liquid chromatography (HPLC, Shimadzu CBM-20A) equipped with a C18 column (150 mm × 2.1 mm, 5 μm). The mobile phase consisted of acetonitrile containing 0.1% formic acid and deionised water (20: 80, V/V) delivered at 1.0 mL/min. All measurements were performed in triplicate and averaged. The equilibrium and kinetic data were interpreted by fitting to the Langmuir and Freundlich models, as well as the pseudo-first-order, Elovich, and intra-particle diffusion kinetic equations [43–45].


$$\begin{array}{c} \text{Langmuir}:\;q_{e}=q_{m}K_{L}c_{e}/(\text{1}+K_{L}c_{e}) \end{array}$$ (1)

$$\begin{array}{c} \text{Freundlich}:\;q_{e}=K_{F}{c_{e}}^{\frac{\text{1}}{n}} \end{array}$$ (2)

In which, q_m_ is the maximum adsorption capacity, mg/g c_e_ is the residual concentration of TMP in the solution at adsorption equilibrium, mg/L. q_e_ is the adsorption capacity of TMP in adsorption equilibrium, mg/g. K_L_ is Langmuir model parameter, L·mg^-1^. K_F_ (mg·g^-1^·L^1/n^·mg^-1/n^) and n are Freundlich model parameters.


$$\begin{array}{c} \text{Pseudo first}-\text{order kinetics}:\;q_{t}=q_{e}[\text{1}-\text{exp}(-k_{\text{1}}t)] \end{array}$$ (3)

$$\begin{array}{c} \text{Elovich kinetic model}:\;q_{t}=\frac{\text{ln}(\alpha \beta )}{\beta }+\frac{\text{ln}t}{\beta } \end{array}$$ (4)

$$\begin{array}{c} \text{Intra}-\text{particle diffusion}:\;q_{\text{e}}=k_{\text{2}}{\text{t}}^{\text{0}.\text{5}}+C \end{array}$$ (5)

In which, q_t_ is the adsorption amount of TMP at t time, mg/g. q_e_ is the adsorption capacity of TMP in adsorption equilibrium, mg/g. k_1_ is the rate constant of pseudo first-order kinetics model, min^-1^. α is the initial adsorption rate of Elovich kinetic model, mg·(g·min)^-1^. β is the desorption constant (or surface-coverage coefficient) of Elovich kinetic model, g/mg. k_2_ is the rate constant of Intra-particle diffusion model, mg·g^-1^·min^-05^.

### 2.6 Dynamic adsorption

Continuous fixed-bed column experiments were conducted to remove TMP from simulated flowing wastewater. A working solution of TMP was prepared at a concentration of 50 mg/L, maintained at 25 °C and pH 7.0. HFRC was packed into the column at different bed heights (0.5, 1.0, and 2.0 cm) and operated under various flow velocities (1, 2, and 5 mL/min) to investigate TMP removal. Breakthrough was defined as the point at which the TMP concentration in the effluent equaled that in the influent, indicating adsorption saturation of the medium. After adsorption was completed, samples were collected from the mixture using a 10 mL syringe, filtered through 0.2 μm membranes, and analyzed in triplicate. The adsorption kinetics were subsequently evaluated using the Thomas and Yoon-Nelson models [46].

The Thomas model incorporates Langmuir-type reversible kinetics, assumes negligible axial dispersion, and employs a second-order rate constant (K_Th_) to quantify the adsorption-desorption equilibrium. Its analytical solution is capable of describing the entire breakthrough curve and is commonly applied to estimate the maximum adsorption capacity (q_e_) under column operating conditions.


$$\begin{array}{c} \text{ln}(\frac{C_{\text{0}}}{C_{\text{t}}}-\text{1})=\frac{K_{Th}q_{Th}\text{hT}}{F}{-\text{K}}_{\text{Th}}{\text{C}}_{\text{0}}\text{t} \end{array}$$ (6)

where C_0_ denotes the influent concentration (mg·L^-1^), C_t_ represents the solution concentration at time t (mg·L^-1^), K_Th_ (mL·mg^-1^·min^-1^) denotes the rate constant, q_Th_ (mg·g^-1^) represents the total adsorption capacity, t (min) is the time, h (cm) is the bed height, T (K) is the absolute temperature, and F (mL·min^-1^) is the flow velocity.

The Yoon-Nelson model simplifies the microscopic adsorption mechanism by using a single rate constant (K_YN_) to describe the probability of adsorbate capture by the adsorbent. It assumes that the breakthrough time (t) follows a symmetric logistic distribution centered on the 50% breakthrough time (τ), making it particularly suitable for rapid prediction of the median breakthrough point.


$$\begin{array}{c} \text{ln}(\frac{C_{\text{t}}}{C_{\text{0}}-C_{\text{t}}})={\text{K}}_{\text{YN}}\left(\text{t}-\tau \right) \end{array}$$ (7)

where t (min) is the duration of the dynamic adsorption process, K_YN_ is the rate constant, and τ (min) is the time required for 50% breakthrough of the bed.

## 3 Results and discussion

### 3.1 Preparation of modified adsorbent

**Fig 2:**
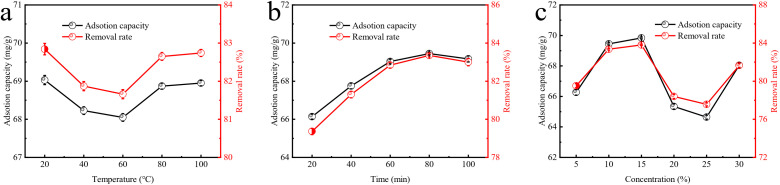
Single-factor test results. **a.** Temperature, **b.** Time, **c.** Concentration.

Fig 2 presents the single-factor experimental results for H_2_O_2_-modified FRC under the following conditions: a TMP concentration of 50 mg/L, pH 7, an adsorbent dosage of 0.030 g, and a contact time of 150 min. When the modification time was fixed at 60 min and the H_2_O_2_ concentration at mass fraction 10%, the adsorption capacity and removal rate of TMP by the modified FRC first decreased and then increased with rising modification temperature. During the initial oxidation stage, H_2_O_2_ preferentially grafts carboxyl, hydroxyl and other acidic oxygen-containing functional groups onto the FRC surface [47], enhancing surface hydrophilicity and facilitating TMP uptake. At 40–60 °C, accelerated H_2_O_2_ decomposition attenuates its oxidizing power. Subsequent to an increase in temperature, the hydrothermal treatment strengthens the modification. Although there is a slight contraction of the specific surface area due to collapse of the pore walls or enlargement of the micropores, there is a marked increase in the density of surface oxygen functionalities [48]. When the modification temperature was maintained at 20 °C and the H_2_O_2_ concentration at 10%, prolonging the modification time caused the TMP adsorption capacity and removal rate to increase initially and then decline. During the moderate-oxidation phase (< 80 min), the progressive introduction of electron-donating groups (-COOH and -OH) intensifies hydrogen bonding and electrostatic interactions between TMP and the modified FRC. Meanwhile, the specific surface area and micropore volume have not yet deteriorated significantly, leading to enhanced adsorption. However, for treatments exceeding 80 min, excessive oxidation erodes the layered carbon scaffold, collapses micropores and triggers decarboxylation, reducing the number of effective adsorption sites and shifting the pore size distribution toward larger pores [49]. Consequently, 80 min represents the optimal modification duration. Finally, at 20 °C and 80 min, increasing the H_2_O_2_ concentration produced a similar rise-and-fall trend in both TMP adsorption capacity and removal efficiency. At low concentrations (< 10%), ·OH radicals generated by H_2_O_2_ incorporate carboxyl, phenolic hydroxyl and carbonyl groups into the carbon matrix. These electrophilic oxygen functionalities establish multipoint cooperative interactions, including hydrogen bonding, Lewis acid-base complexation, and π-π electron donor-acceptor interactions, with the pyrimidine nitrogen and amide carbonyl of TMP. Notably, the microporous structure remains essentially intact, thereby elevating adsorption capacity [50,51]. Once the H_2_O_2_ concentration exceeds the optimum, excessive etching of the carbon skeleton occurs: micropore walls collapse and both the specific surface area and micropore volume decrease sharply, ultimately causing a pronounced reduction in TMP adsorption capacity and removal efficiency.

### 3.2 Characteristics of adsorbents

**Fig 3:**
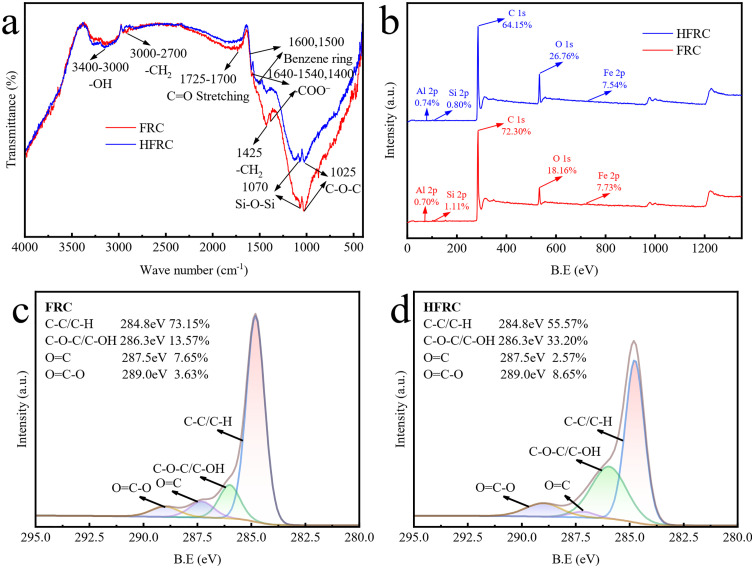
Chemical characteristics of adsorbents. **a.** FTIR spectrum, **b.** XPS full spectrum, Fitting curve of C1s peak of FRC (**c**) and HFRC **(d)**.

**Fig 4:**
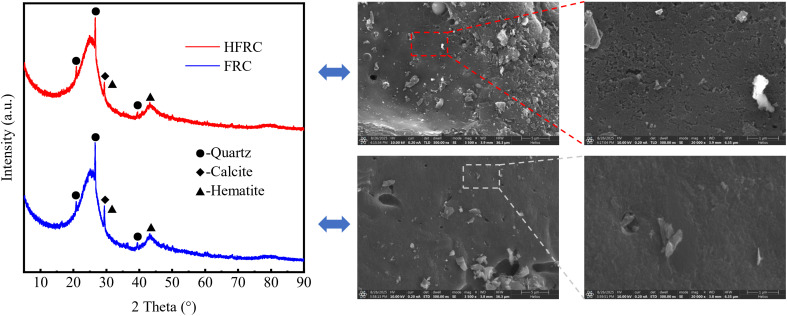
XRD patterns and SEM results of the adsorbent.

**Fig 5:**
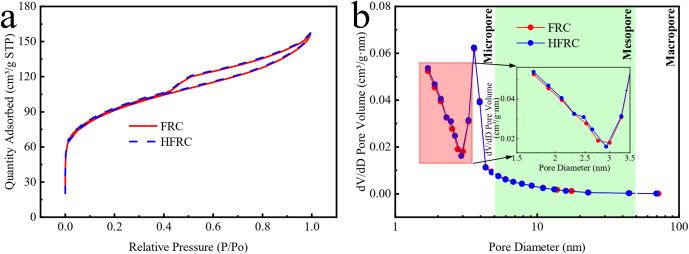
BET results for the adsorbent. **a.** Isotherm linear absolute plot, **b.** Pore size distribution.

The characteristics of the adsorbent are summarized in Figs 3, 4, 5 and Table 3. The data in Fig 3 demonstrate that H_2_O_2_ modification triggers a pronounced reconstruction of the chemical architecture of FRC: the global abundance of surface oxygen-bearing functionalities (-COOH, -OH, etc.) rises markedly, whereas hydrophobic moieties (-CH_2_-) decline commensurately. This evolution originates from the deep oxidation of the carbon scaffold by hydroxyl radicals (·OH) generated during H_2_O_2_ decomposition. FTIR spectroscopic evidence (Fig 3a) reveals a synchronous intensification of absorbance bands at 3400−3000 cm^-1^ and at 1640−1540, 1500 and 1400 cm^-1^, corroborating a substantial increase in alcoholic hydroxyls, aromatic rings and carboxylic hydroxyls. Conversely, the attenuation of bands at 1725−1700 cm^-1^ (C = O stretching) and 1025 cm^-1^ (C-O-C stretching) indicates that a fraction of carbonyl and ether linkages are further oxidised to carboxyl or higher-valence oxygenated species [49]. This oxidative pathway not only elevates surface polarity but also strengthens the affinity of the adsorbent toward the polar molecule TMP. The XPS full spectrum (Fig 3b) indicates a substantial augmentation in the atomic fraction of oxygen on HFRC, thereby validating the experimental findings. The high-resolution C 1s curve-fitting (Fig 3c and 3d) provides a quantitative analysis of the transformation. The relative decrease in the C-C/C-H and O = C- components, as compared to the FRC, is 17.58% and 5.08%, respectively. Conversely, there is an increase in the C-O-C/C-OH and O = C-O components by 19.63% and 5.02%, respectively. The enrichment of oxygenated functionalities, particularly hydroxyl and carboxyl groups, provides a substantial number of polar sites, thereby facilitating efficient TMP uptake.

**Table 3:** BET results for the adsorbent.

Sample	BET surface area (m^2^/g)	Total pore volume (cm^3^/g)	t-plot micropore volume (cm^3^/g)	Average pore diameter (nm)
FRC	329.3634	0.2420	0.0528	2.9384
HFRC	332.0318	0.2434	0.0518	2.9324

XRD patterns (Fig 5) exhibit broad “bread-loaf” humps for both FRC and HFRC within 15–30° 2θ, signifying a predominantly amorphous or micro-crystalline carbon matrix [27]. This carbonaceous phase coexists with crystalline quartz and calcite, implying possible physical encapsulation or chemical bonding between carbon and mineral ash. As a potent oxidant, H_2_O_2_ induces calcite dissolution and promotes the amorphisation of quartz, thereby profoundly altering the surface morphology of FRC. Following H_2_O_2_ treatment, micro-cracks and localized etching appeared on the HFRC surface (Fig 5). To corroborate this observation, BET analysis was conducted (Fig 5 and Table 3). The Isotherm linear absolute plot shifted only marginally, the BET specific surface area rose from 329.3634 m^2^/g to 332.0318 m^2^/g, and the average pore diameter decreased from 2.9384 nm to 2.9324 nm, indicating that the micro-porous architecture of the FRC was altered only slightly. Taken together with the FTIR and XPS results, the enhanced adsorbent performance is ascribed to modifications in surface functional groups rather than to pore diameter changes [52,53].

**Fig 6:**
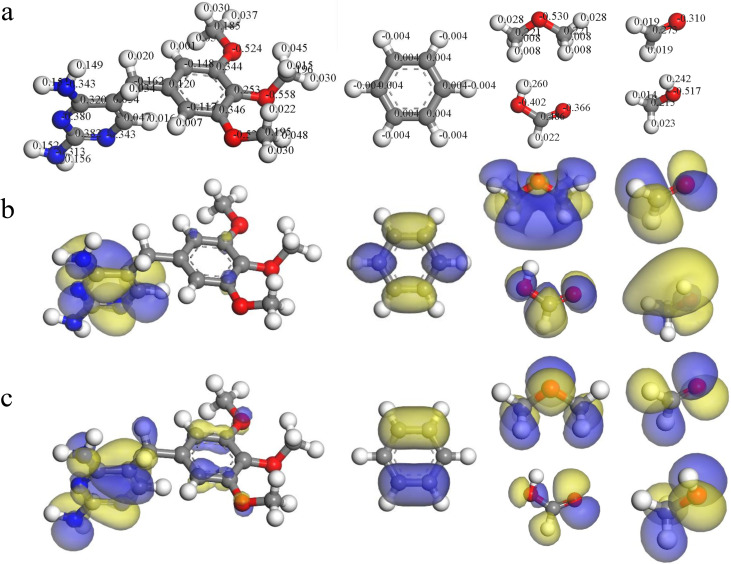
Properties of the molecule. **a.** Charge, **b.** LUMO, **c.** HOMO. (Black, white, red, and blue represent carbon atoms, hydrogen atoms, oxygen atoms, and nitrogen atoms.).

With the advancement of chemical theory and computational technology, the interactions between TMP and various functional groups of the adsorbent can now be understood at the molecular scale, thereby elucidating how H_2_O_2_ induced modifications in the distribution of surface functional groups influence TMP adsorption behavior. The molecular properties of TMP, oxygen-containing functional groups, and benzene rings are illustrated in Fig 6. Charge analysis, performed by calculating and assigning local atomic charges using the Mulliken method, provides critical insights into the electronic structure and physicochemical characteristics of molecules. This approach quantitatively reveals molecular polarity, reactive sites, and the nature and strength of intermolecular interactions, thus bridging the gap between electronic structure and macroscopic properties. The highest occupied molecular orbital (HOMO) and lowest unoccupied molecular orbital (LUMO) are not merely static energy labels but serve as dynamic indicators of molecular reactivity and selectivity in electronic structure theory [54]. The electron density distribution of the HOMO identifies the regions most prone to electron loss, with its energy level directly correlated to ionization potential and nucleophilic activity. Conversely, the nodal topology and energy position of the LUMO indicate the capacity of the molecule to accept electrons, serving as microscopic criteria for evaluating electron affinity and electrophilic reactivity (with blue regions denoting higher activity). Structurally, TMP consists of a 2,4-diaminopyrimidine ring and a 3,4,5-trimethoxybenzyl moiety connected via a methylene bridge. Its functional groups include aromatic amines (-NH_2_), methoxy groups (-OCH_3_), ether linkages (C-O-C), and conjugated π-systems of both the benzene and pyrimidine rings. Negatively charged nitrogen and oxygen atoms constitute the primary contributors to electrostatic interactions and hydrogen bonding between TMP and oxygen-containing functional groups on the adsorbent surface. In terms of van der Waals forces, dispersion interactions dominate, primarily arising from π-π stacking between TMP’s aromatic rings and the graphene planes of activated carbon, with substantial strength attributed to the large contact area. Secondary contributions stem from possible dipole-dipole and induced-dipole interactions between TMP’s polar substituents (methoxy and amino groups) and surface oxygenated groups (e.g., carboxyl and hydroxyl). Moreover, electron sharing between oxygen/nitrogen atoms on the adsorbent surface and TMP molecules has been identified as a key driving mechanism of chemisorption [5,55].

Following H_2_O_2_ modification, the abundance of oxygen-containing functional groups, specific surface area, and exposure of aromatic structures on HFRC were all significantly enhanced compared with the pristine FRC, thereby accelerating TMP adsorption.

**Fig 7:**
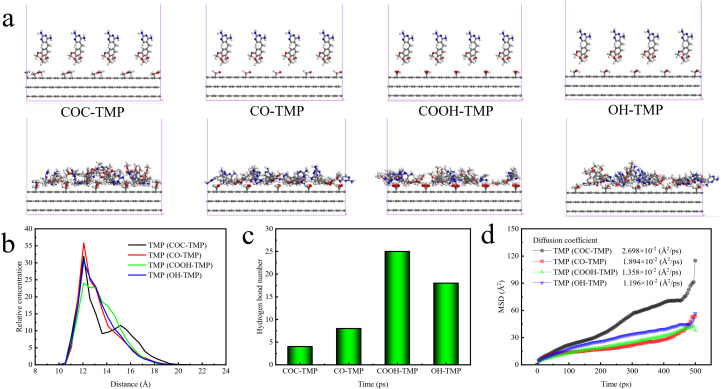
Data Analysis. **a.** Initial and equilibrium conformations, **b.** Relative concentrations, **c.** Number of hydrogen bonds, **d.** Mean square displacement (MSD) and diffusion coefficient.

The initial and equilibrium configurations derived from molecular simulations are presented in Fig 7a. In this study, the adsorption energies of TMP on carbon-based models enriched with oxygen-containing functional groups were systematically calculated, together with their corresponding energy components [56]. The calculated adsorption energies of TMP on surfaces functionalized with C-O-C, C = O, -COOH, and -OH were −341.192, −418.339, −458.620, and −394.311 kJ/mol, respectively. All values were markedly negative, indicating the thermodynamic spontaneity of TMP adsorption on these surfaces (Table 4). The adsorption strength followed the order: -COOH > C = O > -OH > C-O-C, a trend further corroborated by relative concentration profiles (Fig 7b). Comparative experiments confirmed that H_2_O_2_ oxidation introduced concurrent increases in the surface abundance of -COOH, C-O-C, and OH groups on HFRC (Fig 3), thereby resulting in significantly higher adsorption rates than conventional adsorbents [57–60].

**Table 4:** Total interaction energy and energy component of each model.

Model	Interaction energ/(kJ/mol)			
Nonbond energy	Van der Waals	Long-range correction	Electrostatic
C-O-C & TMP	−341.192	−316.156	−3.165	−21.871
C = O & TMP	−418.339	−333.671	−3.068	−81.600
-COOH & TMP	−458.620	−292.199	−3.109	−163.312
-OH & TMP	−394.311	−278.465	−3.062	−112.784

Van der Waals and electrostatic interactions dominated the adsorption process. Van der Waals forces comprise dispersion, dipole-dipole, and induction interactions [61,62]. These forces were notably stronger between TMP and surfaces functionalized with C-O-C and C = O groups than with those featuring -COOH or -OH groups. This effect arose from the synergistic interplay between electron cloud polarizability and interfacial hydrogen-bond competition. C-O-C and C = O groups possess extended π-conjugated frameworks and relatively less electronegative peripheral atoms (C, O), which confer higher static polarizabilities than carboxyl (-COOH) and hydroxyl (-OH) groups. This enhanced polarizability facilitated stronger instantaneous dipole–dipole coupling, thereby contributing additional attractive forces. In contrast, electrostatic interactions were most pronounced for -COOH due to its strong polarity, large dipole moment, and bifunctional capability as both proton donor (-OH) and acceptor (C = O). These properties enabled intense ion-dipole interactions and multiple hydrogen bonds with the electron-rich amino and ether oxygen moieties of TMP [63]. The OH group lacked the dual donor-acceptor synergy of -COOH, while C = O functioned solely as a hydrogen-bond acceptor with weaker interactions. The ether oxygen in C-O-C was delocalized within a conjugated system, exhibiting the lowest polarity and, consequently, the weakest electrostatic interactions [64]. Thus, the order of electrostatic attraction strengths was -COOH > -OH > C = O > C-O-C, with -COOH representing the key group that reinforces interfacial interactions due to its unique acid–base properties and integrated hydrogen-bonding capabilities.

**Fig 8:**
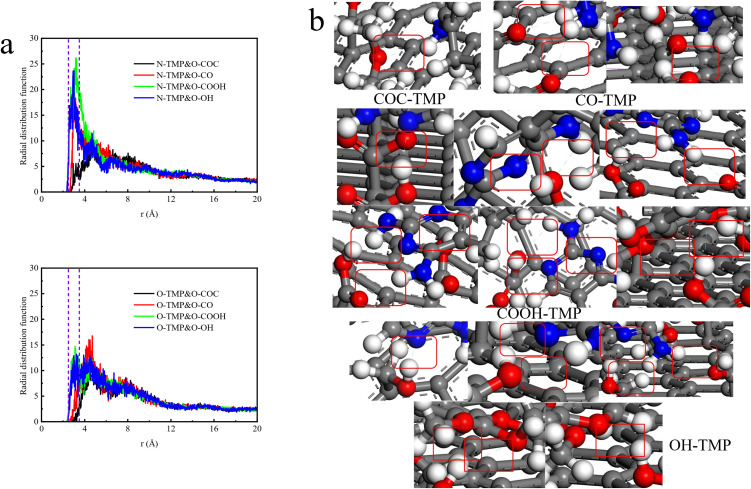
Hydrogen Bond Composition and Configuration. **a.** Radial distribution function, **b.** Configuration.

Hydrogen bonding provided an additional contribution, with TMP forming diverse configurations with different functional groups [65,66]. -COOH could act as both a donor (O-H···N/O) and an acceptor (C = O···H-N), forming strong bifunctional hydrogen bonds with TMP’s amino and ether oxygen groups. OH could serve as a donor (O-H···N/O) or a weak acceptor (O···H-N), resulting in moderate hydrogen-bonding interactions. CO functioned exclusively as an acceptor (C = O···H-N), engaging in single-point interactions with TMP’s amino group. By contrast, C-O-C, lacking active protons and with limited electron lone-pair exposure, acted only as a weak acceptor (C-O-C···H-N), forming relatively unstable hydrogen bonds (Fig 8). Consequently, the order of hydrogen bond numbers was primarily determined by the donor and acceptor capacities of each group: -COOH provided both strong donor (acidic H) and strong acceptor (carbonyl O) sites, -OH offered donor capacity but weaker acceptor ability, C = O acted solely as an acceptor, while C-O-C, constrained by electron delocalization and steric hindrance, exhibited the weakest acceptor ability and lacked any donor function. The resulting hydrogen-bonding sequence was therefore -COOH > -OH > C = O > C-O-C (Fig 7c).

At the microscopic level, the low diffusion coefficients and strong adsorption of TMP on -COOH, -OH, and C = O functionalized surfaces were attributable to the cooperative restrictions imposed by these interactions (Fig 7d). Following H_2_O_2_ oxidation, the increased abundance of C-O-C groups enhanced van der Waals forces, while the concurrent enrichment of -COOH and -OH groups significantly strengthened electrostatic interactions and hydrogen bonding. Collectively, these effects accelerated the adsorption kinetics of TMP through a synergistic mechanism. This structure-property coupling endowed the modified HFRC with superior capacity and faster TMP sequestration compared with unmodified FRC, providing both theoretical insights and practical advantages for efficient environmental remediation.

**Fig 9:**
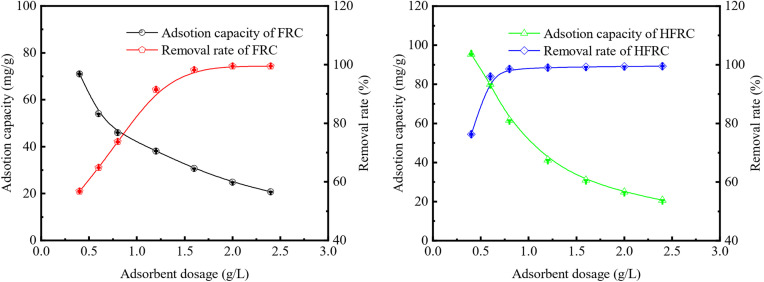
Effect of adsorbent dosage on TMP adsorption.

Fig 9a illustrates the variation in the adsorption performance of FRC and HFRC toward TMP under different dosages. With increasing adsorbent dosage, the adsorption capacity exhibits a gradual decline, whereas the overall removal efficiency increases markedly. This phenomenon can be ascribed to the substantial enlargement of the total specific surface area at higher dosages, which provides a greater number of adsorption sites and thereby enhances the bulk removal of TMP from solution. A further comparison reveals that HFRC possesses a stronger affinity and selectivity for TMP than FRC, indicating that the H_2_O_2_ modification increases the number of active adsorption sites. The dosage corresponding to the intersection of the unit adsorption capacity and removal efficiency curves is commonly regarded as the optimal dosage for the adsorption system [67]. Accordingly, 0.6 g/L was selected for subsequent adsorption experiments.

**Fig 10:**
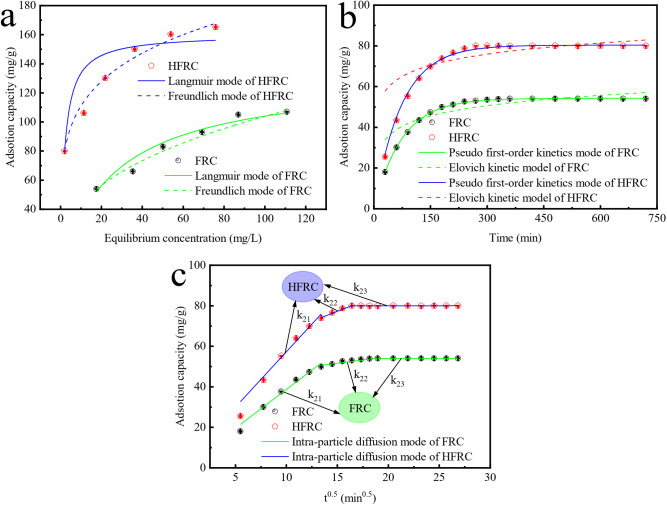
Experimental data and model fitting. **a.** Adsorption models, **b.** Kinetic models, **c.** Intra-particle diffusion.

The effect of the initial TMP concentration on adsorption performance is illustrated in Fig 10a. A progressive enhancement in the uptake of both pollutants is observed as the initial TMP concentration rises, because this concentration constitutes the principal driving force that overcomes the solid-liquid mass-transfer resistance [6]. The adsorption capacity of HFRC exceeds that of the unmodified counterpart, demonstrating that HFRC can rapidly and efficiently eliminate TMP in domains where the local concentration is elevated, and underscoring the superiority of H_2_O_2_ modification. Adsorption isotherms were fitted with established models, and the results are compiled in Table 5. The adsorption isotherm of TMP onto HFRC is better described by the Freundlich model, indicating that the adsorption process involves multilayer or pore-filling behavior rather than ideal monolayer adsorption. Moreover, its relatively high adsorption rate and capacity are attributed to the modifications in surface functional groups induced by the treatment, as well as its intrinsic pore structure.

**Table 5:** Parameters of the fitting curves for various models.

Model types	Constant	FRC	HFRC
Langmuir mode	q_e_ K_L_ R^2^	132.13 0.0369 0.9794	160.29 0.4985 0.9597
Freundlich mode	K_F_ n R^2^	18.0960 0.3794 0.9914	69.5220 0.2035 0.9964
Pseudo-first-order model	q_e_ k_1_ R^2^	54.2113 0.0136 0.9983	80.3437 0.0137 0.9790
Elovich model	α β R^2^	24.9514 0.1369 0.7407	398.0869 0.1268 0.4978
Intra-particle diffusion	k_21_ C_21_ R_21_^2^ k_22_ C_22_ R_22_^2^ k_23_ C_23_ R_23_^2^	3.8093 0.6060 0.9812 0.6438 42.0128 0.9125 0.0011 54.0021 0.1143	5.4565 2.7384 0.9818 1.9512 47.9660 0.9925 0.0019 79.9604 0.1179

Adsorption profiles of TMP uptake by FRC and HFRC, together with the corresponding kinetic fits, are presented in Fig 10b. Equilibrium is attained within 270 min for FRC and 360 min for HFRC, with respective capacities of 54.02 and 80.01 mg/g. The H_2_O_2_-treated FRC evidently accelerates and intensifies TMP sequestration from the aqueous phase. Kinetic modelling (Table 5) reveals that the pseudo-first-order equation best fits the experimental data, indicating that the overall process is governed by physical diffusion and that the rate is proportional to the number of unoccupied surface sites [43]. This outcome corroborates the hypothesis that van der Waals forces, hydrophobic interactions, and π-π stacking dominate TMP adsorption, and implies that the ultimate capacity is constrained by pore architecture and specific surface area. The higher k₁ value obtained for HFRC signifies faster migration of TMP molecules from the bulk liquid to the active surface sites, enabling the system to approach equilibrium more rapidly. Furthermore, the non-equilibrium stages of adsorption on both FRC and HFRC conform to the intra-particle diffusion model: the initial step reflects film diffusion, whereas the second step represents intraparticle transport. The non-zero intercept of the fitted lines evidences the simultaneous operation of film and intra-particle diffusion mechanisms [68,69].

**Fig 11:**
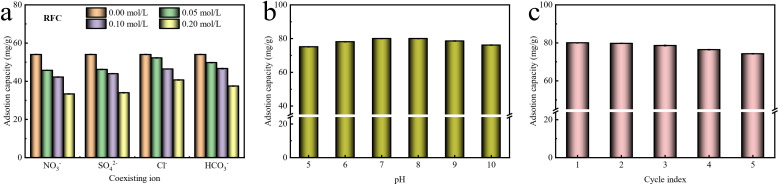
Adaptive characteristics of HFRC. a. Coexisting ion, **b.** pH, **c.** Cycle index.

The H_2_O_2_-modified HFRC exhibits superior adsorption performance relative to the pristine FRC. Consequently, the adaptive characteristics of HFRC were systematically evaluated, and the corresponding results are presented in Fig 11. Investigating sorbent performance across a gradient of ionic strengths furnishes indispensable thermodynamic evidence for full-scale deployment. In typical effluents, NO_3_^-^, SO_4_^2-^, Cl^-^ and HCO_3_^-^ coexist as background electrolytes, among which NO_3_^-^ exerts the most pronounced competitive pressure. HFRC buffers the interference of co-existing anions under elevated ionic strength and retains a high affinity for the target contaminant (Fig 11a). Solution pH, the master variable of aquatic chemistry, governs not only the speciation of the contaminant but also the surface-charge density of the adsorbent via protonation/de-protonation reactions [16]. As shown in Fig 11b, the adsorption capacity of HFRC toward TMP remains virtually constant within the pH window 5–10, evidencing its robustness against wide pH fluctuations encountered in complex water matrices. Reusability is a pivotal criterion for assessing the life-cycle economics of an adsorbent. Five consecutive adsorption-desorption cycles were therefore conducted under static conditions (Fig 11c). Although the equilibrium capacity exhibits an exponential decline with cycle number, the loss after the fifth cycle is only 7.19%, corroborating the synergistic reinforcement of structural integrity and the regenerability of surface active sites in HFRC. Notably, HFRC reaches and 80.01–165.03 mg/g within 270 min, substantially outperforming previously reported sorbents: Microplastics (1.11–1.31 mg/g, 60 h) [70], peanut husk modified with potassium permanganate (25 mg/g, 360 min) [71], modified peanut husk (16–22 mg/g, 180 min) [72], mesoporous silica/reduced graphene oxide composite (17.60 mg/g, 30 min) [73], hydrogel-TiO_2_ (0.10–2.00 mg/g, 18 h) [59], and commercial activated carbon F400 (18.40 mg/g, 10 d) [12]. This comparison underscores the superior sorption capacity and kinetic efficiency of HFRC, attesting to its promise as a next-generation adsorbent.

The production cost of ton-scale HFRC fabricated from CGCS via a flotation-H_2_O_2_ oxidation coupling route, expressed in Ren Min Bi (CNY), is itemized below. In the flotation stage, operated with 16.8 kg/t kerosene and 12 kg/t sec-octyl alcohol at a concentrate yield of 19.13%, 5.23 t of CGCS can be processed to obtain 1 t of FRC. Based on current industrial procurement prices (kerosene 6.3 CNY/kg, sec-octanol 10.0 CNY/kg), the reagent cost amounts to 1,180 CNY. During the oxidation stage, 1 t of FRC consumes approximately 3,000 kg of 27.5% Industrial-grade H_2_O_2_, incurring an expenditure of 1,900 CNY. After adding 10% overhead and profit, the ex-works cost of HFRC is approximately 3,388 CNY/t, which is markedly lower than the prevailing market price of conventional activated carbons (4,000–20,000 CNY/t), demonstrating the pronounced economic advantage of converting CGCS into HFRC.

### 3.5 Dynamic adsorption and model fitting

**Fig 12:**
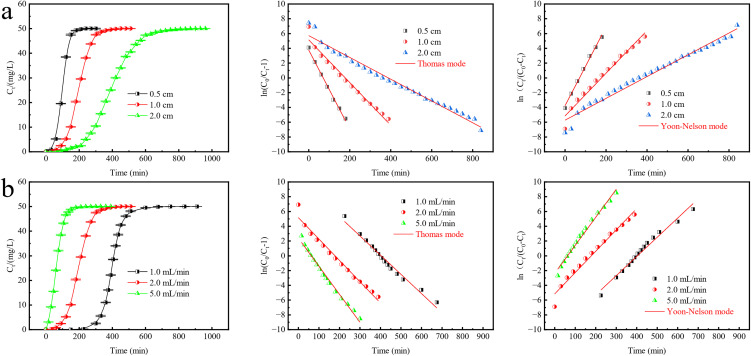
Experimental data and model fitting. **a.** Bed height, **b.** Flow velocity.

The experimental data pertaining to the dynamic adsorption performance of HFRC, along with the corresponding model-fitting outcomes, are systematically summarized in in Fig 12 and Table 6.

**Table 6:** Parameters of the fitting curves for various models.

Mode	Parameters					
Thomas	h cm	F mL/min	T K	q_Th_ mg/g	K_Th_ mL/(mg·min)	R^2^
	0.5	2.0	298	47.60	0.00107	0.9948
	1.0	2.0	298	59.91	0.00058	0.9740
	2.0	2.0	298	65.18	0.00029	0.9780
	1.0	1.0	298	67.69	0.00052	0.9797
	1.0	5.0	298	52.46	0.00076	0.9865
Yoon-Nelson	h cm	F mL/min	T K	τ min	K_YN_ min^-1^	R^2^
	0.5	2.0	298	70.91	0.05340	0.9948
	1.0	2.0	298	178.52	0.02889	0.9740
	2.0	2.0	298	388.45	0.01474	0.9780
	1.0	1.0	298	62.54	0.02601	0.9797
	1.0	5.0	298	403.45	0.03792	0.9865

As shown in Fig 12a, increasing the bed height provides a greater number of accessible adsorption sites. Theoretically, extending the bed height can prolong the operational lifespan of the column, thereby reducing the frequency of regeneration and replacement and consequently lowering labor costs. Breakthrough curves obtained at different bed heights were fitted using the Thomas model, with the fitting parameters summarized in the table. The coefficients of determination (R^2^) for the three experimental sets were 0.9948, 0.9740, and 0.9780, indicating excellent agreement between the model and experimental data. The calculated equilibrium adsorption capacity (q_0_) increased with bed height, reaching 47.60, 59.91, and 65.18 mg/g, respectively. As bed height increased, the adsorption rate constant (K_Th_) decreased monotonically, attributed to the extended solid-liquid contact time and the consequent reduction in mass transfer driving force, whereas the equilibrium adsorption capacity (q_Th_) increased due to longer residence time. Similarly, the Yoon-Nelson model also demonstrated excellent fitting performance (R^2^ > 0.9700). The model parameter K_YN_ decreased with increasing bed height, while the 50% breakthrough time (τ) was correspondingly prolonged, further confirming the effect of contact time.

Fig 12b illustrates that increasing the flow velocity significantly shortens the time required for TMP to reach adsorption equilibrium, which is attributed to limited solid-liquid contact under high flow conditions. Fitting with the Thomas model indicated that K_Th_ increased linearly with flow velocity, consistent with the experimentally observed earlier breakthrough. The corresponding equilibrium adsorption capacities (q_0_) were 67.69, 59.91, and 52.46 mg/g, showing a decreasing trend. Although higher flow rates enhance the mass transfer driving force, they simultaneously reduce the residence time of solutes within the column, leading to decreased utilization of adsorption sites and, consequently, a lower q_Th_. Linear fitting using the Yoon-Nelson model yielded correlation coefficients exceeding 0.9700, confirming good agreement. The adsorption rate constant K_YN_ increased gradually from 0.02601 to 0.03792, reflecting a shorter time required to reach adsorption saturation. With increasing influent flow rate, the residence time of the liquid phase in the fixed bed decreased, resulting in a reduction in the 50% breakthrough time (τ).

## 4 Conclusion

This study employed the green oxidant H_2_O_2_ to modify the surface of CGCS flotation residual carbon and systematically evaluated the adsorption performance of the resulting adsorbent toward a typical antibiotic pollutant TMP. The underlying enhancement mechnism was also thoroughly elucidated. The main conclusions are as follows:

(1) Experimental results indicated that the optimal modification conditions were 20 ℃, 15% H_2_O_2_ concentration, and 80 min treatment time. In these conditions, equilibrium uptakes of TMP by FRC and HFRC reached 54.02 mg/g and 80.01 mg/g, with equilibrium times of 270 min and 360 min, respectively. These results confirm the potential for efficient adsorption.
(2) The adsorption behavior conformed to the Freundlich, pseudo-first-order kinetic, and Intra-particle diffusion models, demonstrating that the adsorption of TMP by HFRC is a rapid process dominated by physical diffusion and jointly governed by film and intra-particle diffusion. H_2_O_2_ modification markedly enlarged both the aromatic domains and the number of reactive oxygen sites, thereby strengthening van der Waals, electrostatic and hydrogen-bonding interactions and ultimately enabling efficient TMP adsorption.
(3) HFRC maintained a pronounced affinity for TMP despite the competitive presence of co-existing anions, and accommodated wide pH fluctuations typical of complex aquatic matrices. After multiple operational cycles, the adsorbent retained >90% of its original adsorption capacity, corroborating its environmental robustness and engineering sustainability.
(4) With increasing bed height, the residence time of TMP molecules on the HFRC surface within the fixed-bed column is substantially prolonged, thereby facilitating the progressive establishment of adsorption equilibrium from a kinetic perspective. Concurrently, the higher bed height offers a greater number of accessible adsorption sites, effectively delaying the onset of the breakthrough curve. In contrast, an elevated flow velocity reduces the contact time between solute and adsorbent, constrains the completeness of the mass transfer-diffusion process, and consequently results in a shorter breakthrough time, decreased treated water volume, and ultimately a lower overall adsorption capacity per unit bed. These findings elucidate the coupled mechanisms between mass transfer and adsorption kinetics in fixed-bed systems, while also providing critical insights for the optimization of bed configuration and operational parameters in engineering practice, thereby offering valuable guidance for the large-scale application of HFRC.

## Supporting information

S1 File Highlights. (DOCX)
