# Supplementary material for: Surface modification of carbon-based adsorbents derived from coal gasification slag for the efficient adsorption of trimethoprim
Source: PLoS One. 2026 Jun 10;21(6):e0351221. doi: 10.1371/journal.pone.0351221 (PMC13252809; doi:10.1371/journal.pone.0351221)
Supplement: S1 File — (DOCX) [file pone.0351221.s001.docx]

1. Residual carbon was treated with H_2_O_2_ for the first time as an adsorbent for TMP.
2. H_2_O_2_ boosts micropores, O-sites, aromatics for multilayer TMP adsorption.
3. Molecular simulations reveal the adsorption process of TMP.
4. Low-cost HFRC merges rapid cleanup with circular-economy slag valorization.
